# A Chinese Child Presented with Early T Cell Precursor Lymphoblastic Lymphoma

**DOI:** 10.1155/2021/5561860

**Published:** 2021-09-28

**Authors:** Xiangyang Pu, Shengyong Deng, Nange Yin, Lin Song, Xiangling He, Jianwen Xiao

**Affiliations:** ^1^Ministry of Education Key Laboratory of Child Development and Disorders, Chongqing, China; ^2^Department of Pediatric Respiratory Medicine, Qianjiang Central Hospital of Chongqing, Chongqing, China; ^3^Department of Pharmacy, Children Hospital of Chongqing Medical University, Chongqing, China; ^4^National Clinical Research Center for Child Health and Disorders, Chongqing, China; ^5^China International Science and Technology Cooperation Base of Child Development and Critical Disorders, Chongqing 400014, China; ^6^Department of Pediatric Hematology and Oncology, Children's Medical Center of Hunan Provincial People's Hospital (The First-Affiliated Hospital of Hunan Normal University), Changsha, China; ^7^Chongqing Key Laboratory of Pediatrics, Chongqing, China; ^8^Department of Hematology, Children Hospital of Chongqing Medical University, Chongqing, China

## Abstract

T cell lymphoblastic lymphoma (T-LBL) is regarded as the leukemic phase of T cell acute lymphoblastic leukemia (T-ALL). The early T cell precursors ALL/LBL (ETP-LBL/ALL) are derived from thymic cells at the ETP differentiation stage and recognized as a high-risk subgroup of T-ALL/LBL. Most of these cases presented with ALL at the disease onset, but the ETP-LBL phase is uncommon. Here, we report a patient who presented with ETP-LBL at the disease onset. In this case, ALL developed even despite receiving chemotherapy, but the patient achieved a complete remission with intensive chemotherapy.

## 1. Introduction

T cell lymphoblastic lymphoma (T-LBL) and T cell acute lymphoblastic leukemia (T-ALL) are both hematological tumors that originate from an immature T cell lineage. T-ALL is regarded as the leukemic phase of T-LBL, and a lymphoblast count in bone marrow (BM) < 25% is defined as the cutoff value between T-LBL and T-ALL [1, 2]. The prognosis of T-LBL/ALL remains historically poor. ALL-based chemotherapy and allogenic hematopoietic stem cell transplantation (allo-HSCT) demonstrated effective results, and event-free survival (EFS) in children and adolescents exceeds 80–90% in developed countries [2, 3]. However, the survival of relapsed and/or refractory cases remains poor [2–4]. The early T cell precursors ALL/LBL (ETP-LBL/ALL) are derived from thymic cells at the ETP differentiation stage and are recognized as a high-risk subgroup of T-ALL/LBL [5]. ETP-LBL/ALL occurs in 10–12% or 20–25% of pediatric or adult T-LBL/ALL populations [5–7]. Most of these cases present with ALL, and the LBL phase is uncommon [6–8]. Here, we report a patient who presented with ETP-LBL at disease onset. ALL developed eventhough he subsequently received chemotherapy, and he achieved a complete remission (CR) with intensive chemotherapy.

## 2. Case Presentation

**Figure 1:**
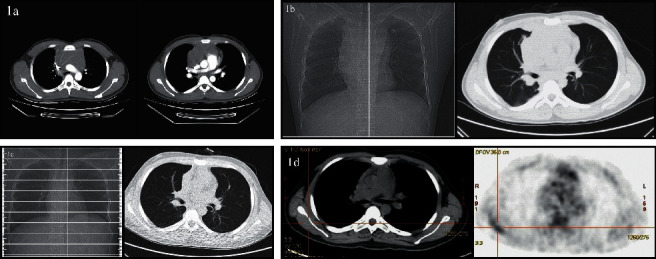
Results of chest CT or PET-CT scan.

**Figure 2:**
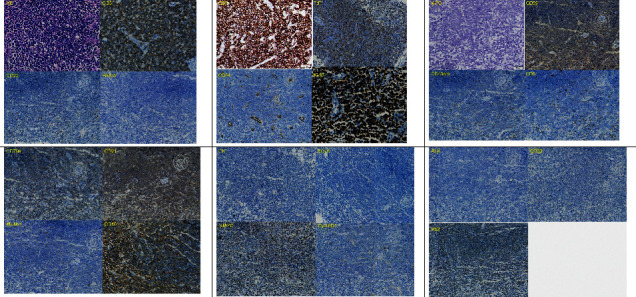
Results of mediastinum mass sample.

**Figure 3:**
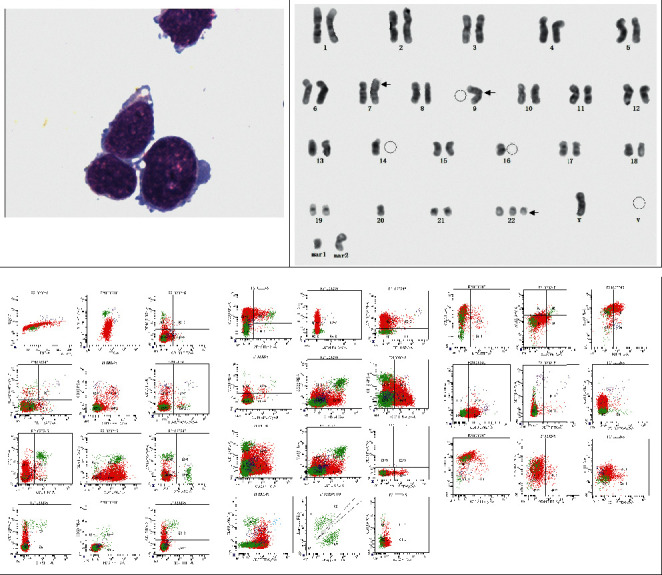
Results of pleural effusion sample.

A 16-year-3-month-old boy was admitted with an irritating dry cough and cervical lymphadenopathy on 28 January 2020. During the physical examination, bilateral cervical lymph node masses were palpable, and signs of pleural effusion were also noted. Laboratory tests showed normal complete blood cell counts (white blood cells 6.22 × 10^9^/L, platelets 295 × 10^9^/L, and hemoglobin 161 g/L) and normal lactate dehydrogenase levels (233 U/L). A computed tomography (CT) scan revealed pericardial and right pleural effusion and lymphadenopathy at the cervical area bilaterally and within the mediastinum (Figure 1(a)). A core needle biopsy and subsequent immunohistochemical (IHC) staining of the mediastinum mass was performed for CD3+, CD20−, PAX5−, CD7+, CD34−, Ki-67+(90%), MPO−, and CD99+(Figure 2). A sample of the pleural effusion was analyzed which showed that the nucleated cell counts were 31 × 10^9^/L, the lymphoblasts were 92%, and there were 55.4% T cell lymphoblasts as detected by flow cytometry (FCM). Positive results for CD7, CD13, and cCD3 and negative results for CD1a, CD4, CD8, CD19, CD20, CD22, CD34, and MPO were confirmed (Figure 3). A diagnosis of ETP-ALL/LBL was considered, and additional IHC samples of the mediastinum mass sample were stained via IHC and were tested. The results showed CD45pro-,CD5-, and CD79a were weak+, CD21 was positive, MUM-1 was negative, CD10 was positive, CK was negative, BCL6 was negative, c-MYC was (40%+), CyclinD1 was negative, ALK was negative, CD30 was negative, and P53 was negative (Figure 2).

A chromosome karyotype of the pleural effusion sample was obtained using the International System of Human Cytogenetic Nomenclature 2009 (ISCN-2009) [2]: 43–50, *X*, *Y*, add(7) (p13), 9,?i(9) (q10), 14, 16, +22,?del(22) (q13), inc[CP8] (Figure 3). BM samples were acquired at the bilateral posterior superior iliac area, and blast cells were not detected by a BM smear, biopsy, or via FCM.

Samples were taken from the mediastinum mass, and whole exome sequencing (WES) and RNA sequencing (RNAseq) were performed. FBXW7 and GATA3 somatic mutations were detectable by WES (Table 1); transcripts of FBXW7, GATA3, JAK1, and NCOR1 were confirmed by RNAseq (Table 2).

**Figure 4:**
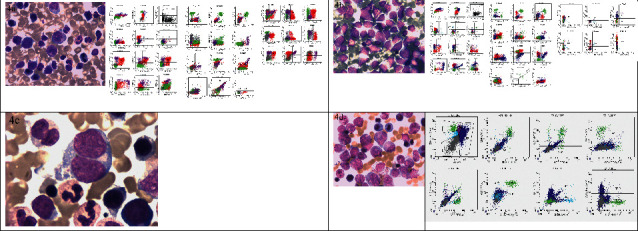
Results of bone marrow samples.

A diagnosis of ETP-LBL was given based on previous literature reports [5–8], and the patient was classified as stage III by the revised International Pediatric Non-Hodgkin's Lymphoma Staging System [9]. He was treated with a modified BFM-LBL-95 protocol and was placed in an intermediate-risk (IR) group at the initial diagnosis [10]; the detail of the disease evaluation, risk group classification, treatment courses, dosages, and intrathecal injections had been listed at Supplementary Tables S1–S5, respectively; his treatment response was evaluated by BM, CT, or positron emission tomography/computed tomography (PET/CT) imaging at different time points (TP) as the protocol required. On day 15 and day 33 (TP1 or TP2) during the course of the induction of remission I, the BM smear showed 29.5% and 80% blast cells, FCM had 29% and 55.4% ETP lymphoblasts (Figures 4(a) and 4(b)), and a chest CT scan revealed an unchanged mediastinum mass (Figure 1(b)).

He was considered to have progressive disease (PD) and refractory disease at TP2 [5, 6], and two standard courses A of the HyperCVAD protocol [11] were administered. After the 1st course, lymphoblasts were no longer detected in the BM during the evaluation of the BM smear (Figure 4(c)); the CT scan showed a partial remission (PR) [5, 6], which was subsequently monitored by CT scans (Figure 1(c)). He achieved CR after the 2nd course A, which was monitored by using BM smears (Figure 4(d)) and PET-CT scans (Figure 1(d)). The minimal residual disease (MRD) level was also detected by FCM [12], and a negative result was obtained (<10^−4^). Allo-HSCT was declined by the family, and he was treated with the CCLG-ALL-2008 protocol for the high-risk (HR) group [12]. Up until June 2021, he was alive without any evidence of a relapse.

## 3. Discussion

ETP-ALL/LBL is a recently described subgroup of T-ALL/LBL according to the World Health Organization criteria from 2016 (WHO 2016) [4]; usually, these cases are aggressive and present with ETP-ALL, whereas pediatric ETP-LBL is uncommon (20–30% in adult T-ALL/LBL, pediatric data are absent). In the presented case, we reported a child who suffered from ETP-LBL without BM infiltration at the onset.

ETP-ALL/LBL patients are distinguished from non-ETPs by FCM and genomic signatures. ETP-ALL/LBL is immunophenotypically deﬁned by weak or absent expression of T cell markers (CD1a, CD5, and CD8) and positive expression of at least one hematopoietic stem cell (HSC) and/or myeloid markers (CD13, CD33, CD34, CD117, and HLA-DR). The diagnosis of T-LBL was based on the pathological results and IHC staining, but not all the T cell, HSC, and myeloid markers were evaluated. Fortunately, the patient was diagnosed with ETP-ALL/LBL by FCM detected in a pleural effusion sample. FCM is an important technique to subclassify T-ALL/LBL, and all these T-ALL/LBL samples should be evaluated if possible.

Genomic signatures of ETP-ALL/LBL patients also exhibit unique genotypes compared with those of non-ETPs patients [7, 8]. Recurrent mutations were not only identified in the genes involved in T-lymphoid development or oncogenesis because myeloid markers were also detectable. Activating mutations encoding cytokine receptors and mediating the RAS signal transduction system (NRAS, KRAS, FLT3, and JAK1), inactivating mutations encoding transcription factors (GATA3, ETV6, and RUNX1) and histone repair (EZH2 and EP300) during hematopoietic stem cell development are common in ETP-ALL/LBL patients [5, 6, 13]. WES and RNAseq of LN samples have been evaluated, common gene mutations and transcripts (FBXW7, NOTCH1, and JAK1) in T-ALL/LBL have been identified, and unique genomic signatures of ETP-ALL/LBL have also been identified. This suggests that gene analysis can assist in the diagnosis of ETPs without using FCM detection.

The best treatment and the prognosis for ETPs patients is unclear. Treatment responses and outcomes for ETP-ALL/LBL patients treated with chemotherapy were poorer than those for non-ETP-ALL/LBL adult patients, and it was reported that these cases benefit from allo-HSCT [4–6]. Although early reports of pediatric ETP-ALL showed a poor prognosis, recent clinical trials of COGAALL0434 and UKALL2003 showed that the prognosis of ETP-ALL was similar to that of other T-ALLs [14, 15]. However, ETP-LBL was uncommon, but the treatment experience was limited. The patient in this case report was treated with a modified BFM-LBL-95 protocol, the ALL-based classic protocol for T-LBL patients, but he was considered to have refractory disease and infiltration into the BM. It was reported that adults benefited from the hyperCVAD protocol [5, 6], and in our patient, two courses A were administered, but course B was omitted due to financial constraints. The patient obtained CR, and chemotherapy was continued.

In conclusion, we reported a case of ETP-LBL that took an aggressive clinical course. Our case revealed that clinicians should pay attention to the possibility of ETP-LBL in common T-LBL cases, and FCM and genomic signatures are important to distinguish ETP-LBL. Further studies are needed to research the most effective treatment for ETP-ALL/LBL patients.

## Figures and Tables

**Table 1:** Results of whole exome sequencing.

Genes	Chromosome coordinate	Mutation site	Mutation frequency (%)
FBXW7 (NM_033632)	chr4: 153247366	c.1436G > A (p.Arg479Gln)	38.3
GATA3 (NM_0010022)	chr10: 8106004	c.827G > A (p.Arg276Gln)	38.8
GATA3 (NM_0010022)	chr10: 8105986	c.811_812dup (p.Thr272ArgfsTer24)	26.7

**Table 2:** Results of RNA sequencing.

Gene mutations	Mutation type	Mutation frequency (%)
FBXW7 R479Q	Missense mutation	51.9
GATA3 R276Q	Missense mutation	68.8
GATA3 T270fs	Frameshift mutation	24.4
JAK1 L783F	Missense mutation	58.5
NCOR1 Y1435^*∗*^	Truncating mutation	34.6
NOTCH1 V1721E	Missense mutation	34.1

## Data Availability

The data used to support the findings of this study are included within the article.

## Supplementary Materials

Supplementary Materials Table S1: disease evaluation. Table S2: risk group. Table S3: schedule of the protocol. Table S4: modified NHL-LBL-95 protocol. Table S5: schedule of intrathecal injections.
