# Supplementary material for: A Chinese Child Presented with Early T Cell Precursor Lymphoblastic Lymphoma
Source: Case Rep Hematol. 2021 Sep 28;2021:5561860. doi: 10.1155/2021/5561860 (PMC8492265; doi:10.1155/2021/5561860)
Supplement: Supplementary Materials — Table S1: disease evaluation. Table S2: risk group. Table S3: schedule of the protocol. Table S4: modified NHL-LBL-95 protocol. Table S5: schedule of intrathecal injections. [file 5561860.f1.docx]

**Table-S1 Disease evaluation**

|  | Definition |
| --- | --- |
| CR | Disappearance of all tumor evidence, included physical examinaion, tests of BM, CSF and CT/PET-CT scan; biospy of suscept LN showed no tumor evidence. |
| PR | Size of tumor reduced ＞50%, new lesion or progressive lesion was undetected, CNS or BM infiltation was not detected. |
| PD | Progress or new lesions on the basis of the original disease status. |
| Relapsed | New evidence of tumor after patients got CR |
| CR: Complete remission; PR: Partial remission; PD: Progressive disease | |

**Table-S2 Risk group**

| Risk group | Definition |
| --- | --- |
| SR | Stage I or stage II |
| IR | Stage III or stage IV |
| HR | PR or RD at TP2, CR at TP3 |
| RD | PR, RD or PD at TP3 |
| SR: standard risk; IR: intermediate risk; HR: high risk; RD: Refractory disease | |

**Table-S3 Schedule of the protocol**

| Risk group | Induction I | Consolidation | Induction II | maintenance treatment | Total |
| --- | --- | --- | --- | --- | --- |
| SR | VDLP+CAM1-2 | HD-MTX×4 |  | 6-MP+MTX+VD | Male:2.5year  Female:2year |
| IR | VDLP+CAM1-2 | HD-MTX×4 | VALDex+CAM | 6-MP+MTX+VD |  |
| HR |  | HR1-3×2 |  | 6-MP+MTX+VD+CA |  |

**Table -S4 Modified NHL-LBL-95 protocol**

| Phase | Drug | Dosage | Duration |
| --- | --- | --- | --- |
| Induction Ia | Pred | 60 mg/m^2^/d, po | D1-28, tapering after 9 days |
|  | DNR | 30 mg/m^2^/d, iv, 1h | D1, 8, 15, and 22 |
|  | VCR | 1.5 mg/m^2^/d (max: 2 mg) iv | D1, 8, 15, and 22 |
|  | L-asp^&^ | 8000 U/m^2^/d, iv | D8, 11, 13, 15, 17, 19, 21, 23, 25 and 27 |
|  | CTX | 1 g/m^2^/d, iv, 1h | D36 |
|  | AraC | 75 mg/m^2^/d , iv | D38-41, 45-48 |
|  | 6-MP | 60 mg/m^2^/d, po | D36-39 |
|  | TiT | Related to age | D1,8,15,22 and 45 |
| Induction Ib | CTX | 1 g/m^2^/d, iv, 1h | D1 |
|  | AraC | 75 mg/m^2^/d , iv | D3-6,10-13 |
|  | 6-MP | 60 mg/m^2^/d, po | D1-14 |
|  | TiT | Related to age | D10 |
| Consolidation (SR or IR) | MTX^#^ | 5 g/m^2^/d, iv | Days 1, 15, 29, and 43 |
|  | 6-MP | 25 mg/m^2^/d, po | D1-56 |
|  | TiT | Related to age | Days 1, 15, 29, and 43 |
| Consolidation (HR or refractory) Block1-3x2 | |  |  |
| Block1 | Dex | 20 mg/m^2^ iv | D1-5 |
|  | VCR | 1.5mg/m^2^ iv | D1, 6 |
|  | MTX^#^ | 5g/m2 iv 24h | D1 |
|  | CTX | 200mg/m2 iv 1h, q12h | D2-4 (5 dose) |
|  | AraC | 2g/m^2^ iv q12h，3h | D5 |
|  | L-Asp | 25000U/ m^2^ iv 3h | D6, 11 |
|  | TiT | Related to age | D1 |
| Block2 | Dex | 20 mg/m^2^ iv | D1-5 |
|  | VDS | 3mg/m^2^ (max: 5mg) iv 3h | D1, 6 |
|  | MTX^#^ | 5g/m2 iv 24h | D1 |
|  | IFO | 800mg/m2 iv q12h, 1h | D2-4 (5 dose) |
|  | DNR | 30 mg/m^2^ iv 1h | D5 |
|  | L-asp | 25000U/ m^2^ iv 3h | D6, 11 |
|  | TiT | Related to age | D1 |
| Block3 | Dex | 20 mg/m^2^ iv | D1-5 |
|  | AraC | 2g/m2 iv q12h，3h | D1,2 |
|  | VP-16 | 100mg/m2 iv q12h, 3h | D3-5 (5 dose) |
|  | L-asp | 25000U/ m^2^ iv 3h | D6, 11 |
|  | TiT | Related to age | D1 |
| Induction IIa | Dex | 8 mg/m^2^/d, po or iv | D1-21, tapering after 9 days |
|  | VCR | 1.5mg/m^2^ iv | D1, 8, 15 and 22 |
|  | L-asp | 10000 U/m^2^/d, iv, 3h | D1, 4, 7, 11 |
|  | ADM | 30mg/m^2^, iv, 1h | D1, 8, 15 and 22 (HR group: day 1 and 15) |
|  | TiT | Related to age | D1, 8 |
| Induction IIb | CTX | 1 g/m^2^/d, iv, 1h | D1 |
|  | AraC | 75 mg/m^2^/d , iv | D3-6,10-13 |
|  | 6-MP | 60 mg/m^2^/d, po | D1-14 |
|  | TiT | Related to age | D3, 10 |
|  |  |  |  |
| Maintenance |  |  |  |
| SR or IR group: 10 cycles | MTX | 20 mg/m^2^/d, po | D1, 8, 15, 22,29,36,43,50 |
|  | 6-MP | 25 mg/m^2^/d, po | D1-56 |
|  | VCR | 1.5 mg/m^2^/d (max: 2 mg) iv | D1 |
|  | DEX | 8 mg/m^2^/d, po | D1-7 |
|  | TiT | Related to age | D1 (course 1-5) |
| HR group |  |  |  |
| Course-1 (12 cycles): every 4 weeks | MTX | 25 mg/m^2^/d, po | Days 1, 8, 15, 22 |
|  | 6-MP | 50 mg/m^2^/d, po | Days 1 to 28 |
|  | CTX | 300 mg/m^2^/d, iv | Day 15 |
|  | VCR | 1.5 mg/m^2^/d (max: 2 mg) iv | Day 15 |
|  | AraC | 300 mg/m^2^/d, iv | Day 15 |
|  | DEX | 8 mg/m^2^/d, po | Days 15 to 21 |
|  | TiT | Related to age | Day 15 |
| Course-2 (male: 8 cycles; female: 5cycles) | MTX | 25 mg/m^2^/d, po | Days 1,8,15,22 |
|  | 6-MP | 50 mg/m^2^/d, po | Days 1 to 28 |
|  | VCR | 1.5 mg/m^2^/d (max: 2 mg) iv | Day 15 |
|  | DEX | 8 mg/m^2^/d, po | Days 15 to 21 |
| Course-3 (male: 3 cycles) | MTX | 25 mg/m^2^/d, po | Days 1,8,15,22 |
|  | 6-MP | 50 mg/m^2^/d, po | Days 1 to 28 |

#: Methotrexate is iv 24 h, administration of tetrahydrofolate begins at 42 h after the methotrexate, serum level of the methotrexate is monitored at 48 h, and tetrahydrofolate is withdrawn until the serum level of methotrexate ≤0.5μmol/ml. &: L-asp was replaced by PEG-Lasp (2000U/m^2^).

**Table-S5 Schedule of intrathecal injections**

| Age | Methotrexate | Cytarabine | Dexamethasone |
| --- | --- | --- | --- |
| <12 m | 6 mg | 15 mg | 2.5 mg |
| 12-36 m | 9 mg | 25 mg | 2.5 mg |
| ≥ 36 m | 12 mg | 30 mg | 5 mg |
